# Case Report: A case report of bullous pemphigoid triggered by sintilimab (PD-1 inhibitor)

**DOI:** 10.3389/fimmu.2026.1776044

**Published:** 2026-07-01

**Authors:** Chang Qin, Chuan Yang, Yuqi Wang, Ye Zou, Xiaojie Ding

**Affiliations:** 1 Department of Dermatology, Affiliated Hospital of North Sichuan Medical College, Nanchong, Sichuan, China; 2 Department of Clinical Medicine, North Sichuan Medical College, Nanchong, Sichuan, China

**Keywords:** bullous pemphigoid, case report, cutaneous immune-related adverse events, direct immunofluorescence, immune checkpoint inhibitor, PD-1 inhibitor, sintilimab

## Abstract

Immune checkpoint inhibitors (ICIs) can precipitate autoimmune bullous diseases, among which bullous pemphigoid (BP) is uncommon but has significant clinical implications. We report a 72−year−old man with poorly differentiated gastric adenocarcinoma and liver metastases who developed generalized pruritic erythema after the 4th cycle of sintilimab, followed by tense blisters after the 8th cycle. Upon admission (January 2025), over 30% of his body surface area (BSA) was affected, accompanied by pruritus that impacted his sleep. Histopathology showed eosinophilic cell infiltration within the epidermis and mild spongiosis. Edema was observed between collagen fibers in the papillary dermis, accompanied by a small number of lymphocytes and histiocytes. Moderate (+++) mixed inflammatory cell infiltration was observed in the superficial dermis, primarily consisting of lymphocytes (approximately 60%) and eosinophils (approximately 30%), with a small number of neutrophils (approximately 10%). Direct immunofluorescence (DIF) revealed IgG and C3 along the basement membrane zone (BMZ). Serum autoantibodies demonstrated significantly elevated BP180 (392.01 RU/mL) and low BP230 (9.29 RU/mL), while desmoglein−1 (8.38 U/mL) and desmoglein−3 (5 U/mL) were negative/low, supporting BP rather than pemphigus. The rash met the CTCAE (Common Terminology Criteria for Adverse Events) v5.0 criteria for Bullous dermatitis, Grade 3, with concomitant Pruritus, Grade 3. Daily intravenous methylprednisolone 60 mg was initiated, leading to cessation of new blister formation within 7 days and improvement in pruritus. Sintilimab was discontinued during treatment and resumed after BP was brought under control (approximately 1 month later); the patient has maintained remission for more than 12 months since resuming Sintilimab treatment, and no recurrence has been observed. The temporal association with PD−1 blockade, objective clinicopathologic confirmation, and lack of strong alternative culprits support a possible causal relationship. Early biopsy, DIF, and targeted serology enable timely CTCAE−guided management and informed decision-making for pausing or rechallenging PD−1 therapy.

## Introduction

Cutaneous immune−related adverse events (irAEs) are among the most common toxicities associated with PD−1/PD−L1 blockade (1). BP is an uncommon but clinically significant irAE that, if unrecognized, may jeopardize the continuity of cancer treatment. Diagnosis relies on a clinicopathologic triad: compatible histology, DIF showing linear IgG/C3 along the BMZ, and serologic detection of BP180/230 antibodies; when available, salt−split indirect immunofluorescence (IIF) further localizes the binding (2). We report a case of BP associated with sintilimab, Grade−3 severity, which responded rapidly to corticosteroids, emphasizing the importance of objective confirmation (high−titer BP180) and practical CTCAE−guided management considerations. We highlight a successful PD−1 inhibitor rechallenge after grade−3 BP control, adding practical insights to the emerging spectrum of sintilimab−associated bullous pemphigoid.

## Case presentation

A 72−year−old male with poorly differentiated gastric adenocarcinoma and multiple liver metastases (stage IV) began SOX (S-1 plus oxaliplatin) chemotherapy in April 2024 (Oxaliplatin 200 mg on day 1; Tegafur/Gimeracil/Oteracil 50 mg twice daily on days 1-14; every 3 weeks). Three weeks later, sintilimab was added at 200 mg every 3 weeks. After the 4th cycle (June 2024), he developed generalized pruritic erythema and papules. By the 8th cycle (November 2024), tense vesicles/blisters (ranging from 0.2 cm to 2.0 cm in diameter) appeared on the erythematous background, primarily on the extremities, accompanied by headaches and nocturnal pruritus affecting his sleep. He did not seek treatment. In January 2025, the patient developed a generalized rash while receiving treatment in the oncology department. Therefore, a consultation with dermatology was requested. Based on the clinical presentation, the patient exhibited pruritic tense bullae on an erythematous base, which had a clear temporal relationship with PD−1 inhibitor treatment, strongly suggesting a working diagnosis of BP induced by targeted therapy. Consequently, the patient was referred to our dermatology department for further histopathological and immunopathological evaluation. The patient denied any history of hypertension, infectious diseases, or genetic disorders.

**Figure 1:**
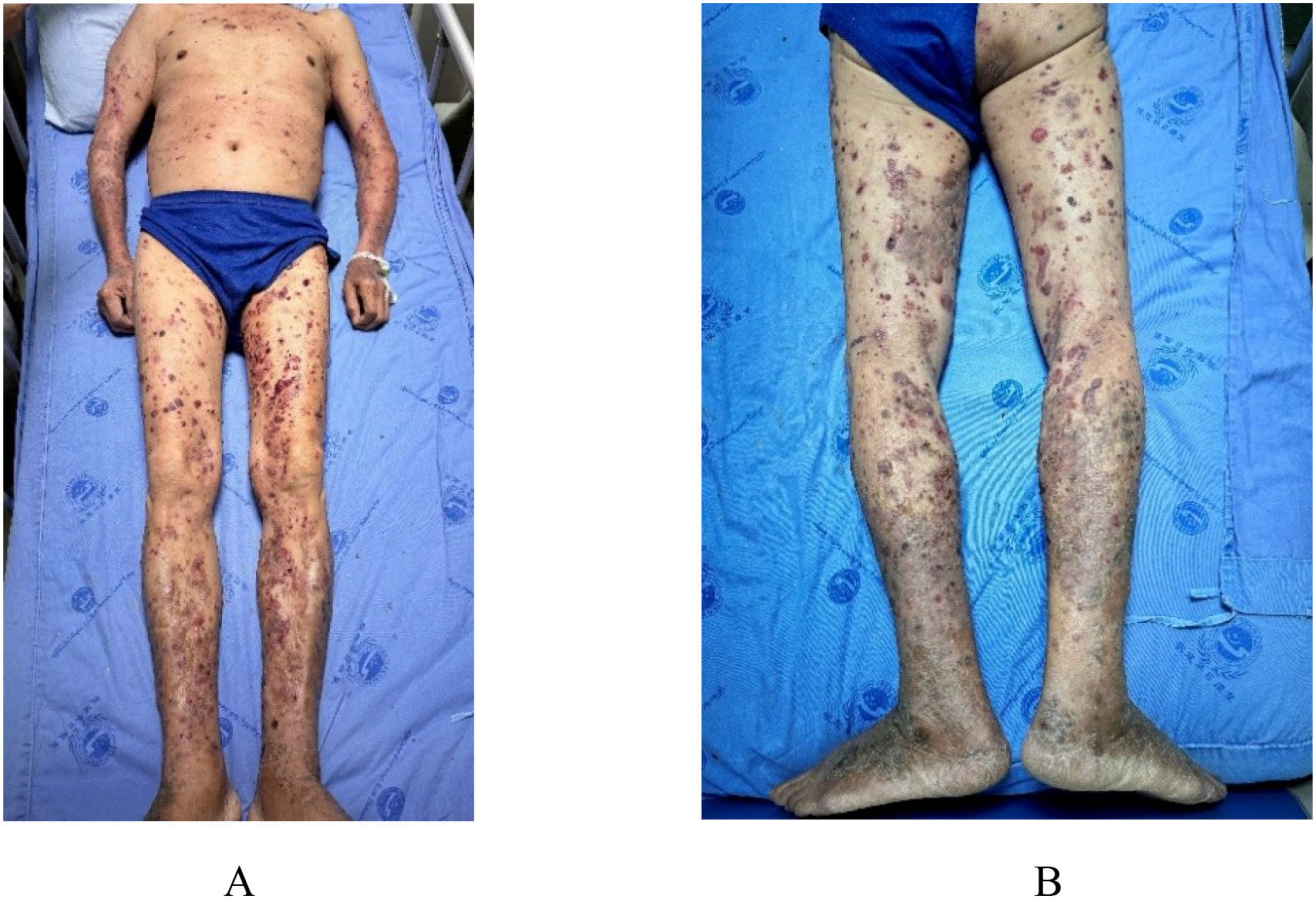
Imaging presentation of the patient. **(A, B)** On the trunk and limbs, the skin showed erythema with scattered to densely distributed round to oval vesicles and bullae, ranging from 0.2 to 2.0 cm in diameter. The blister walls are tense, the blister fluid is clear, and Nikolsky’s sign is negative. Some blisters are hemorrhagic, and after rupture, they undergo erosion and crusting. The toes on both feet are thickened and appear grayish-yellow.

The admission examination revealed extensive involvement of the trunk and limbs, accompanied by erythema, erosion, and multiple tense blisters; estimated BSA >30% (**Figure 1**). There were scratch injuries, consistent with severe pruritus. Mucous membranes were not affected. Vital signs were stable.

**Figure 2:**
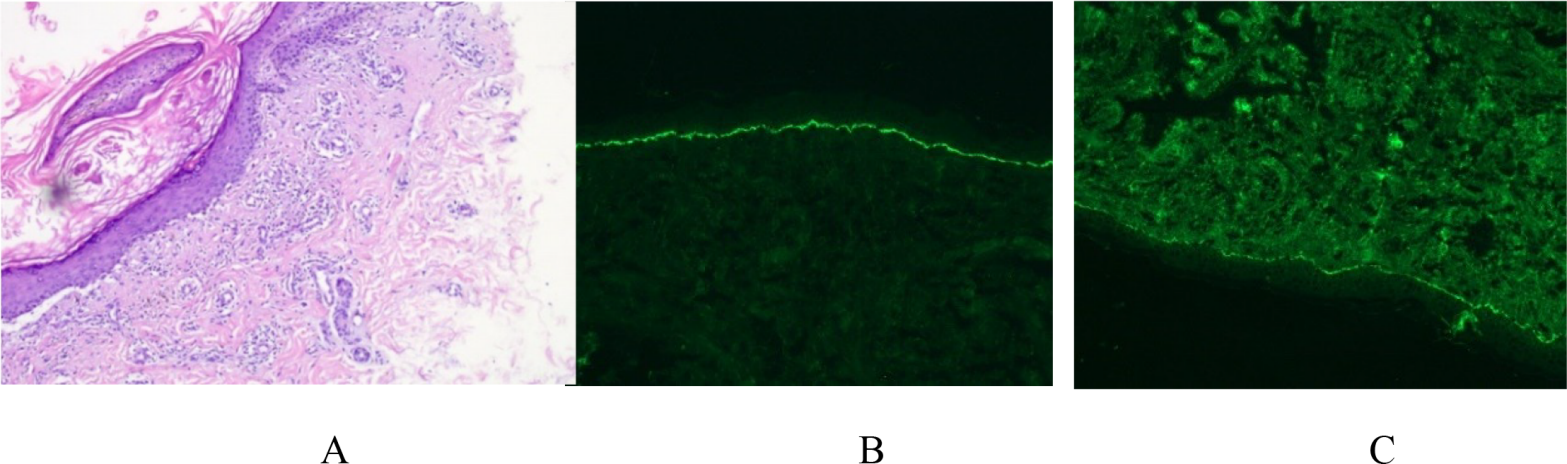
Examination. **(A)** Histopathology of the skin lesions showed eosinophilic cell infiltration within the epidermis and mild spongiosis. Edema was observed between collagen fibers in the papillary dermis, accompanied by a small number of lymphocytes and histiocytes. Moderate (+++) mixed inflammatory cell infiltration was observed in the superficial dermis, primarily consisting of lymphocytes (approximately 60%) and eosinophils (approximately 30%), with a small number of neutrophils (approximately 10%). (HE staining, original magnification, ×100) **(B, C)** Linear deposition of C3 and IgG along the basement membrane, with IgM, IgA, and fibrin all negative. (Direct immunofluorescence, ×40).

Histopathology (lesional skin) demonstrated eosinophilic cell infiltration within the epidermis and mild spongiosis. Edema was observed between collagen fibers in the papillary dermis, accompanied by a small number of lymphocytes and histiocytes. Moderate (+++) mixed inflammatory cell infiltration was observed in the superficial dermis, primarily consisting of lymphocytes (approximately 60%) and eosinophils (approximately 30%), with a small number of neutrophils (approximately 10%) (**Figure 2A**). DIF showed linear deposition of IgG and C3 along the BMZ (**Figures 2B, C**). Serological testing revealed that the level of anti-BP180 antibodies had increased to 392.01 RU/mL (reference range: <20 RU/mL), while the level of anti-BP230 antibodies remained within the normal range at 9.29 U/mL (reference range: <20 RU/mL). The measured levels of demyelinating protein 3 antibodies were 5 U/mL, while those of desmoglein-1 were 8.38 U/mL (reference range: <20 U/mL; both negative/low). The above findings supported a diagnosis of BP rather than pemphigus.

According to NCI CTCAE v5.0, the peak severity corresponds to Bullous dermatitis, Grade 3 (BSA >30% with functional impact), accompanied by Pruritus, Grade 3 (sleep disruption) (3).

Management and early results: The patient weighed approximately 75 kg. Upon admission, intravenous methylprednisolone 60 mg was initiated once daily (equivalent to prednisone 1 mg/kg/day). This steroid dosage is consistent with the recommendations of ASCO (the American Society of Clinical Oncology) 2021 and ESMO (the European Society for Medical Oncology) 2022 guidelines (4, 5). Within seven days, no new blisters formed, and the pruritus improved, indicating a rapid response to steroids. High-potency topical steroids and wound care were applied to the affected areas. After one week, the dose of methylprednisolone was reduced to 40 mg daily for three days. Once the patient’s condition was confirmed to be well-controlled, discharge was advised with regular follow-ups, gradually reducing the steroid dosage. After discharge, the patient switched to oral methylprednisolone 40 mg, in combination with minocycline (100 mg twice daily) and nicotinamide (500 mg three times daily), and began a gradual tapering plan. The complete tapering period from the initial dose to discontinuation was approximately one and a half years. The tapering plan was as follows: a reduction of 10 mg weekly in the early stage, a reduction of 5 mg every two to four weeks in the mid-stage, and finally a reduction of approximately 2.5 mg every two to three months until discontinuation.

## Discussion

Immune checkpoint inhibitor-related bullous pemphigoid (ICI−BP), although uncommon, is receiving increasing attention. This case illustrates several clinically significant features:

1. The importance of successful ICI rechallenge in treatment decisions: In most reported cases of BP associated with sintilimab (**Table 1**), ICIs were permanently discontinued after the occurrence of BP. In contrast, our patient successfully resumed sintilimab therapy after the moderate−to−severe (CTCAE Grade 3) ICI−BP was effectively controlled with corticosteroids. After rechallenge, the patient maintained tumor remission for over 12 months without BP recurrence. This experience provides important clinical reference for managing ICI−BP: under strict safety standards, ICI rechallenge is feasible and may offer sustained benefits to patients.
2. Rapid corticosteroid response (high sensitivity): In our case, intravenous methylprednisolone at 60 mg/day (approximately 1 mg/kg/day) achieved clinical remission within just one week, with no new blisters forming. This response speed is significantly faster than most reports of ICI−BP cases in the literature (which typically require 2–4 weeks). Our case suggests that for elderly patients with poor physical condition, administering a moderate dose of systemic corticosteroids (consistent with ASCO and ESMO guidelines recommending 1 mg/kg/day or 0.5-1.0 mg/kg/day) can rapidly control the disease and create opportunities for early ICI rechallenge after careful risk-benefit assessment.
3. Successful corticosteroid−sparing strategies and combination therapy: In our case, minocycline and nicotinamide were added to the treatment regimen. This combination not only met the clinical needs for infection control and adjunctive anti−inflammatory effects in the treatment of BP, but also supported the reduction of corticosteroids, effectively decreasing the cumulative steroid dosage and treatment duration. This strategy has particular clinical significance for elderly patients with poor physical conditions.
4. A systematic literature review shows that among all reported cases of BP related to sintilimab, this case is notable for successfully re-challenging ICIs after experiencing Grade 3 irAEs, leading to the cessation of corticosteroids and sustained tumor remission (**Table 1**).

**Table 1:** A summary of representative cases of BP associated with sintilimab reported to date.

Author and year	Type of tumor	Time of onset	Treatment regimen	ICI decision	Outcome
He et al. (16)	Renal Cell Carcinoma	About 24 months	Intravenous methylprednisolone 80 mg/day + minocycline + nicotinamide	Stop using ICI	After 4 weeks of treatment, no new lesions appeared
Wang et al. (17)	pMMR/MSS colorectal cancer	6 months	Oral low-dose methylprednisolone maintenance therapy (4 mg twice daily)	Continue using ICI	Partial response > August, no new lesions
Cui et al. (18)	lung squamous cell carcinoma	16 months	Intravenous methylprednisolone 60 mg/day → Oral prednisone, tapering until discontinued	Not reported	No recurrence at 19 months of follow-up
Su et al. (19)	colon cancer	18 months	Intravenous methylprednisolone → Oral prednisone maintenance therapy after 2 months	Resume treatment after BP is controlled(about 2 months)	Remission maintained for >12 months after Resume treatment; no recurrence
In this case	Poorly differentiated gastric adenocarcinoma	4.5 months	Intravenous methylprednisolone 60 mg/day + minocycline + nicotinamide → Oral corticosteroids tapered gradually until discontinued	Resume treatment after BP is controlled(about 1 month)	Remission maintained for >12 months after Resume treatment; no recurrence

The mechanisms by which PD-1 inhibitors induce BP have not been fully elucidated, but may involve the following pathways:

1. Loss of peripheral tolerance: Under steady-state conditions, peripheral immune cells may express PD-1 on their surface. PD-1 inhibitors suppress the expression of PD-1, thereby releasing the inhibition on cells such as CD8+ tissue-resident memory T cells (TRM), CD4+ TRM cells, γδ T cells, innate lymphoid cells (ILC), and natural killer (NK) cells. This leads to the release of cytokines and promotes the infiltration of effector cells (e.g., eosinophils) into the upper dermis, ultimately contributing to the development of BP (6)..
2. T-cell overactivation: PD-1 inhibitors can alleviate the suppression of T-cell proliferation and reduce the promotion of regulatory T-cell (Treg) generation. While enhancing anti-tumor immunity, they may inadvertently activate autoreactive T-cell clones directed against normal tissue antigens, leading to the activation of autoreactive T cells (7, 8). This leads to the activation of T-cell clones specific for BMZ autoantigens such as BP180 and BP230.
3. B-cell activation: The excessive activation of T cells may further promote the activation of B-cells and the production of autoantibodies. These autoantibodies bind to self-antigens (e.g., BP180 and BP230), thereby promoting the development of BP (9)..

These mechanisms together constitute the pathophysiological basis for PD-1 inhibitors breaking immune tolerance and inducing BP.

The diagnostic importance of DIF remains crucial in BP, with linear IgG and C3 deposition at the BMZ being its hallmark (10). High−titer BP180 adds serologic specificity and helps distinguish BP from pemphigus when desmoglein−1/3 is negative or low (11). When available, salt−split IIF can further localize epidermal (“roof”) binding and help exclude epidermolysis bullosa acquisita (EBA, “floor” binding) (12). The lack of salt−split IIF in this case is a limitation of the immunological examination. However, the following reasons ensure diagnostic certainty and reliably exclude EBA: 1) ELISA shows anti−BP180 antibodies significantly above the positive threshold (>20 RU/mL), while the target antigen in EBA is type VII collagen, and anti−BP180 antibodies should not be positive in EBA; 2) The clinical presentation lacks the characteristic features of EBA (skin fragility, atrophic scarring, milia); 3) EBA typically responds poorly to corticosteroid therapy, whereas our patient showed a rapid and effective treatment response. In our patient, even without IIF, the combination of DIF and serology provided strong confirmation. The diagnosis of BP is usually based on the integration of three key elements: a) typical clinical presentation (pruritic tense bullae with negative Nikolsky sign); b) histopathology (subepidermal blister with eosinophil infiltration); c) immunopathological evidence (linear IgG/C3 deposition along the BMZ on DIF and/or detection of anti−BP180 antibodies by ELISA. In this case, all three elements are satisfied, so a clear diagnosis of BP can be made even without performing IIF.

From a management perspective, we advocate a stepwise approach based on CTCAE. For Grade 2 disease, high−potency topical therapy with or without a short systemic corticosteroid taper may permit continuation or early re−initiation of PD−1 once toxicity improves to ≤ Grade 1 under close dermatologic supervision. For Grade 3, consideration may be given to pausing PD−1 inhibitors and combining with systemic corticosteroids (e.g., methylprednisolone 0.5-1.0 mg/kg/day), followed by structured tapering; the option of steroid−sparing agents (such as doxycycline/niacinamide; for refractory cases, biologics like dupilumab or omalizumab, or IVIG) may reduce cumulative steroid exposure in elderly patients with multiple comorbidities (5, 13). In this case, methylprednisolone 60 mg/day achieved cessation of new blisters within a week, supporting steroid responsiveness and the feasibility of rapid disease control.

Importantly, decisions regarding the continuity of oncology (pause vs. continuation vs. rechallenge) should be personalized in a multidisciplinary setting, taking into account cancer response, the severity of irAE, and patient preferences. Notably, in the context of concomitant SOX chemotherapy, temporal relationship analysis strongly points to sintilimab as the primary causative agent. According to the Naranjo algorithm, the score for sintilimab is 5 (Probable), while the score for SOX chemotherapy is only 1 (Possible) (**Table 2**) (14). Moreover, SOX drugs do not belong to the known drug categories that induce BP (such as diuretics, ACE inhibitors, antibiotics, DPP−4 inhibitors, etc.) (15). Therefore, the potential contribution of SOX chemotherapy is more likely to reflect non−specific modulation of the overall immune microenvironment rather than a direct causal trigger.

**Table 2:** The Naranjo adverse drug reaction (ADR) probability scale questionnaire in the patient of this case.

Question number	Sintilimab	SOX	Scoring criteria
1. Are there previous conclusive reports on this reaction?	+1	0	Cases of BP associated with sintilimab have been reported in the past; no such reports have been found in the literature regarding the SOX chemotherapy.
2. Did the adverse event appear after the suspected drug was administered?	+2	+2	The rash appeared after using these two medications.
3. Did the adverse event improve when the drug was discontinued, or a specific antagonist was administered?	+1	0	Symptoms improved after discontinuing sintilimab and undergoing hormone therapy; the SOX chemotherapy was continued.
4. Did the adverse event reappear when the drug was re-administered?	0	0	Re-activation has not been performed; evaluation is not possible.
5. Are there alternative causes that could, on their own, have caused the reaction?	0	-1	Sintilimab is a known drug that can induce BP; according to the literature, it is the most likely cause of BP in this case. The association between the SOX chemotherapy and BP has not yet been established; therefore, it cannot be definitively identified as the cause of BP, but its potential contribution (such as nonspecific immunomodulation) cannot be completely ruled out.
6. Did the reaction reappear when a placebo was given?	0	0	Not completed
7. Was the drug detected in the blood or other fluids in concentrations known to be toxic?	0	0	Not detected
8. Was the reaction more severe when the dose was increased or less severe when the dose was decreased?	0	0	Not evaluated
9. Did the patient have a similar reaction to the same or similar drugs in any previous exposure?	0	0	None
10. Was the adverse event confirmed by any objective evidence?	+1	0	Elevated anti-BP180 antibody levels and positive DIF
Score	5	1	

The ADR is assigned to a probability category from the total score as follows: definite if the overall score is 9 or greater, probable for a score of 5–8, possible for 1–4, and doubtful if the score is 0. The scale uses 10 standardized questions to quantitatively assess the causal relationship between a drug and an adverse reaction; each question is assigned a score of -1, 0, + 1, or +2 based on the response (14).

Limitations include the lack of salt−splitting IIF and the inability to obtain standardized pruritus/BPDAI trajectories. Future prospective registries are needed to refine the safety of PD−1 continuation or rechallenge after ICI−BP and to formalize algorithms for steroid−sparing therapy.

## Conclusion

Sintilimab can trigger clinically significant BP, which has been confirmed through concordant histology, DIF, and high−titer BP180. Early recognition and CTCAE−guided management allow for rapid control in Grade 3 presentations, using systemic corticosteroids. A structured multidisciplinary approach aids in timely treatment while preserving options for oncologic continuity.

## Data Availability

The original contributions presented in the study are included in the article/supplementary material. Further inquiries can be directed to the corresponding author.
